# Error in Article Information

**DOI:** 10.1001/jamanetworkopen.2026.27139

**Published:** 2026-07-09

In the Original Investigation titled “Early Neurodevelopmental Outcomes in Children With Congenital Heart Disease,”^1^ published June 1, 2026, there was an error in the Article Information section. The Funding/Support section should have read: “This project was supported by the Cardiac Neurodevelopmental Outcome Collaborative (CNOC), the Pediatric Cardiac Critical Care Consortium (PC^4^), and the Children’s Heart Foundation through their support for integrated registry initiatives as a part of Cardiac Networks United.” This article has been corrected.^1^
